# TreeRipper web application: towards a fully automated optical tree recognition software

**DOI:** 10.1186/1471-2105-12-178

**Published:** 2011-05-20

**Authors:** Joseph Hughes

**Affiliations:** 1 IBAHCM, College of Medical, Veterinary and Life Sciences, University of Glasgow, Graham Kerr Building, University Avenue, Glasgow, G12 8QQ, UK

## Abstract

**Background:**

Relationships between species, genes and genomes have been printed as trees for over a century. Whilst this may have been the best format for exchanging and sharing phylogenetic hypotheses during the 20^th ^century, the worldwide web now provides faster and automated ways of transferring and sharing phylogenetic knowledge. However, novel software is needed to defrost these published phylogenies for the 21^st ^century.

**Results:**

TreeRipper is a simple website for the fully-automated recognition of multifurcating phylogenetic trees (http://linnaeus.zoology.gla.ac.uk/~jhughes/treeripper/). The program accepts a range of input image formats (PNG, JPG/JPEG or GIF). The underlying command line c++ program follows a number of cleaning steps to detect lines, remove node labels, patch-up broken lines and corners and detect line edges. The edge contour is then determined to detect the branch length, tip label positions and the topology of the tree. Optical Character Recognition (OCR) is used to convert the tip labels into text with the freely available tesseract-ocr software. 32% of images meeting the prerequisites for TreeRipper were successfully recognised, the largest tree had 115 leaves.

**Conclusions:**

Despite the diversity of ways phylogenies have been illustrated making the design of a fully automated tree recognition software difficult, TreeRipper is a step towards automating the digitization of past phylogenies. We also provide a dataset of 100 tree images and associated tree files for training and/or benchmarking future software. TreeRipper is an open source project licensed under the GNU General Public Licence v3.

## Background

**Figure 1:**
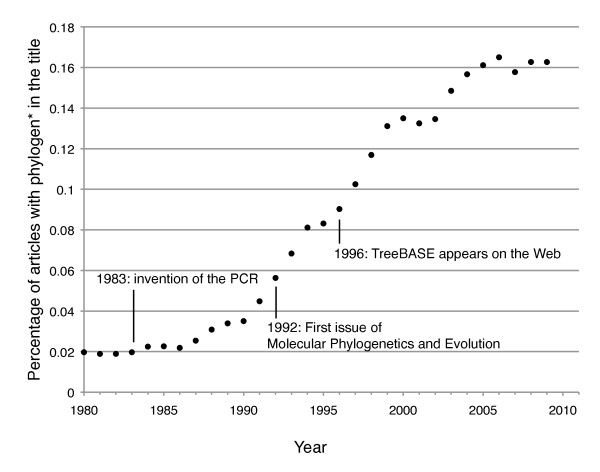
Percentage of articles with phylogen* in the title. The percentage of articles with phylogen* in the title out of the total number of publication for each year since 1980 from PubMed.

In 1859, Darwin produced one of the first illustrations of a phylogenetic tree, notably this was the only figure included in *The Origin of Species *[1]. Since, biologists have used trees to depict the relationships between organisms, genes and genomes. The number of studies depicting phylogenies exploded (see Figure 1) with the development of the polymerase chain reaction technique and journals were created specifically for publishing the molecular phylogenies generated by researchers (e.g., Molecular Phylogenetics and Evolution established in 1992). Whilst in the early years of morphological and molecular phylogenetics, embedding illustrations into manuscripts might have been the most appropriate way to disseminate knowledge, this has resulted in the locking up of phylogenetic hypotheses into the pages of journals and books without an easy way to access this information.

Currently, the construction of the relationships between the 1.8 million currently estimated species largely depends on the unprecedented growth of molecular sequence data [2] and this makes GenBank the most accessible source of comparative data for most taxa in the tree of life [3]. Whilst more sequence data, more powerful computers and improved phylogenetic reconstruction algorithms will enable researcher to generate up-to-date phylogenies from the raw data in the future, past phylogenetic inferences will remain central to guiding researchers towards studying poorly supported relationships and under-sampled lineages. They are also central for studying the effects of new phylogenetic methodologies and new and larger datasets [2].

Not all phylogenetically informative data are confined to sequence databases. TreeBASE is a very valuable repository as it holds morphological or genetic data with the associated published phylogeny [4]. However, as few publishers require submission to TreeBASE as a pre-requisite for publication, a large number of phylogenies remain embedded as images in published articles. Indeed, the rapid growth of published phylogenies is not matched by the availability of those trees in databases (see Figure 1 in [5]).

The idea of using a program to convert a tree image into a computer-readable representation of that tree was first implemented in TreeThief [6] which required the user to trace a tree by clicking on each of its nodes in turn. The latter program is only available for the discontinued operating system Mac OS 9. Laubach and von Haeseler [7] provided a conceptual advance with a semi-automatic program called TreeSnatcher that has recently been updated [8]. TreeSnatcher uses image-processing methods to prepare a tree image and detect the tree structure, it works on rectangular and freeform trees (e.g., radial and star). The user supervises the tree recognition process by making corrections to the image. For example, the user can modify the image in order to make the foreground dark and background light, fill gaps in lines and identify the foreground. The program then determines inner node and tip locations. The user can add or remove further nodes and delete or add branches. The user is then required to assign species names to the tips before the program can build the Newick tree code.

Here, we will review the way researchers present their phylogenies, demonstrate the feasibility of a fully automated tree recognition software and provide a dataset of tree images and associated tree files for training and/or benchmarking future programs.

## Implementation

The current version of TreeRipper opens tree-image files in the formats PNG, JPG/JPEG, or GIF.

• The tree needs to have the root on the left and leaves on the right.

• Horizontal branches.

• The tree constitutes a dark foreground on a light homogenous background (no background boxes or shading).

• The tree must be bi- or multifurcating (not a network)

• The inner nodes are branching points between lines and have no circles, rectangles, etc. inscribed.

• Tip branches must have branch lengths greater than 0.

**Figure 2:**
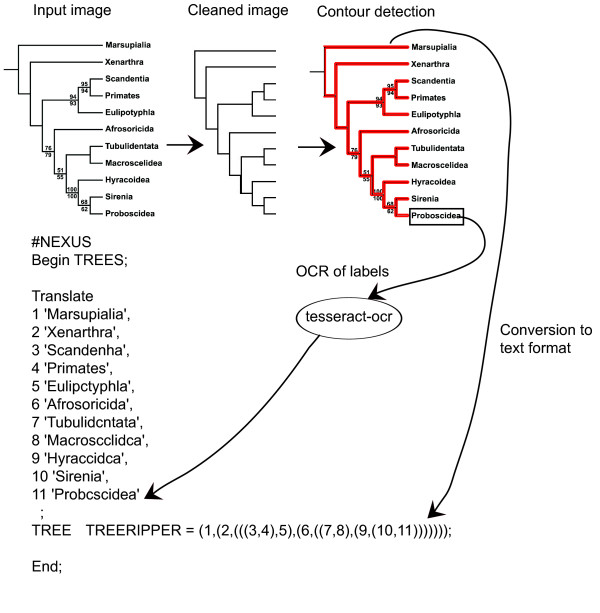
**Architecture of the software design for TreeRipper**. The input image is scaled, node labels are removed, branches are smoothed and corners patched-up, the contour is detected. Tips locations are used to determine leaf label boxes for which the text is recognised using Tesseract. TreeRipper summarizes the tree topology and labels in a text file and an SVG file, which shows the contours.

TreeRipper is written in c++ using a set of Standard Template Library algorithms provided by Magick++. The image is first converted to black and white and rescaled so that horizontal lines are on average 2 pixels thick. The image is cleaned by removing a series of patterns such as black pixels surrounded by a box of white pixels and horizontal lines that are not connected to vertical lines. Lines and corners are then patched up before the contour is traced and the topology detected. The locations of branch tips are then used to crop the tip labels from the original image. Tip labels are converted to text using the freely available tesseract-ocr program. The steps in the program are depicted in Figure 2. The web application written in PHP enables the visualization of the tracing and allows editing of the labels.

## Results and Discussion

**Figure 3:**
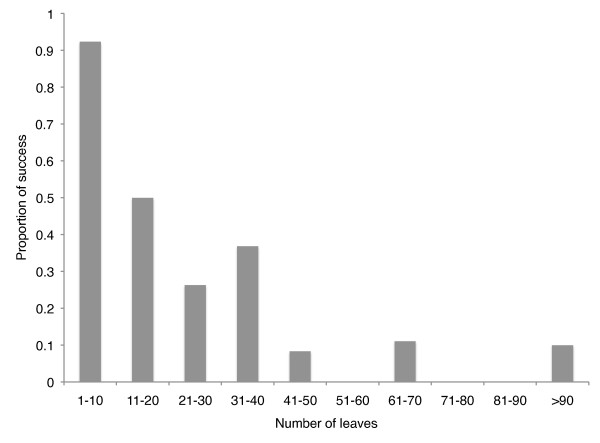
**Proportion of images successfully recognised**. The proportion of tree images successfully recognised by TreeRipper according to the number of leaves on the phylogeny.

We downloaded 322 images which had phylogen* or supertree in their caption from 249 articles published in the Open Access journal BMC Evolutionary Biology between 1997 and 2009. Only eleven out of these 249 articles have submitted their alignment and tree files to TreeBASE. All images were visually inspected to check whether the image met the prerequisites. Twenty-four images were not phylogenies, 26 were represented as radial tree layouts, 8 as polar tree layouts and 5 as cladograms. Of those represented with a rectangular tree layout, 40 had background boxes, 31 had lines intersecting branches or branches drawn with dotted or dashed lines, 32 had circles or boxes as nodes, 6 were illustrated over multiple pages, 4 had triangles as tip leaves, 3 had leaves with zero branch lengths. A further 29 would need some form of pre-processing (rotating or splitting into component images). Of the 298 images of phylogenies downloaded only 114 (38%) met the prerequisites for this program, which are very similar to those of the original semi-automatic recognition software TreeSnatcher [7]. This small proportion of the total phylogenetic images illustrates the plethora of ways trees are currently represented in one journal alone. Of the 114 phylogenies that meet the prerequisites, the topologies of 37 trees (i.e., 32%) were successfully recognized by TreeRipper without any prior processing. The proportion of successfully recognised images was higher for phylogenies with fewer leaves (Figure 3) and the largest phylogeny successfully recognised had 115 leaves. The average processing time was 127 seconds (ranging from 4 to 562 seconds) using a MacBook Pro (2.4 GHz Intel Core 2 Duo with 2 GB 667 MHz DDR2 SDRAM). We do not review the accuracy of the OCR here as it has been done elsewhere (see [9]).

The successfully recognised tree images along with a further 63 images manually converted to tree files are provided as supplementary material in NEXUS, Newick and phyloXML formats [10] (Additional file 1) for training and/or benchmarking future programs.

## Conclusions

Although the program has a high failure rate, it is the first step towards an automated approach for optical tree recognition and proves the feasibility of an approach, which will allow us to defrost published phylogenetic hypotheses. We are unlikely to ever be able to create an application that recognises all possible trees due to the sheer diversity of ways phylogenies have been illustrated but at the very least, this program could be used for automating tree recognition of large sets of tree images before using manual conversion or semi-automated programs like TreeSnatcher for the trees that were not converted.

As phylogenetics enters a third phase of growth with the advent of next-generation sequencing, one hopes that the work of future phylogenetists will be published in a format that will enable the digital curation and preservation of their hard work.

## Availability and requirements

Project name: TreeRipper (automated phylogeny recognition from images)

Webserver: http://linnaeus.zoology.gla.ac.uk/~jhughes/treeripper

Project home page: https://code.google.com/p/treeripper/

Programming language: C++ and PHP web interface

License: GNU GPL v3

### Prerequisites

Tesseract-OCR licensed with the Apache 2.0 License except the tesseractTrainer.py, which is licensed with GPL: http://code.google.com/p/tesseract-ocr

Imagemagick, license is compatible with the GPL: http://www.imagemagick.org/

## Authors' contributions

JH developed the idea, wrote the code, tested the software and drafted the manuscript.

## Supplementary Material

Additional file 1 **Tree images, associated newick file and example Perl script for batch processing**. Set of images and associated nexus tree file as a zip file. Click here for file

## References

[B1] Darwin CR On the origin of species by means of natural selection, or the preservation of favoured races in the struggle for life 1859 1 London: John Murray PMC5184128 30164232

[B2] Smith SA Beaulieu JM Donoghue MJ Mega-phylogeny approach for comparative biology: an alternative to supertree and supermatrix approaches BMC evolutionary biology 2009 9 37 10.1186/1471-2148-9-37 19210768 PMC2645364

[B3] McMahon MM Sanderson MJ Phylogenetic supermatrix analysis of GenBank sequences from 2228 papilionoid legumes Systematic biology 2006 55 818 836 10.1080/10635150600999150 17060202

[B4] TreeBASE: a database of phylogenetic knowledge http://www.treebase.org/

[B5] Page RDM Towards a Taxonomically Intelligent Phylogenetic Nature Precedings 2007 1 5

[B6] TreeThief: a tool for manual phylogenetic tree entry http://microbe.bio.indiana.edu:7131/soft/iubionew/molbio/evolution/phylo/TreeThief/main.html

[B7] Laubach T von Haeseler A TreeSnatcher: coding trees from images Bioinformatics (Oxford, England) 2007 23 3384 3385 10.1093/bioinformatics/btm438 17893085

[B8] TreeSnatcher Plus: a phylogenetic tree capturing tool http://www.cibiv.at/software/treesnatcher/

[B9] Smith R An Overview of the Tesseract OCR Engine Ninth International Conference on Document Analysis and Recognition (ICDAR 2007) 2007 2 629 633

[B10] Han MV Zmasek CM phyloXML: XML for evolutionary biology and comparative genomics BMC Bioinformatics 2009 10 356 10.1186/1471-2105-10-356 19860910 PMC2774328

